# Salivary Redox Biomarkers in Different Stages of Dementia Severity

**DOI:** 10.3390/jcm8060840

**Published:** 2019-06-12

**Authors:** Anna Klimiuk, Mateusz Maciejczyk, Magdalena Choromańska, Katarzyna Fejfer, Napoleon Waszkiewicz, Anna Zalewska

**Affiliations:** 1 Department of Restorative Dentistry, Medical University of Bialystok, 15-437 Bialystok, Poland; annak04@poczta.onet.pl (A.K.); choromanska100@gmail.com (M.C.); ksawicka1@wp.pl (K.F.); azalewska426@gmail.com (A.Z.); 2 Department of Physiology, Medical University of Bialystok, 15-437 Bialystok, Poland; 3 Department of Psychiatry, Medical University of Bialystok, 15-437 Bialystok, Poland; napoleonwas@yahoo.com

**Keywords:** oxidative stress, antioxidants, saliva, redox biomarkers, dementia

## Abstract

This study is the first to evaluate oxidative stress biomarkers in saliva/blood of patients with varying degrees of dementia progression. The study included 50 healthy controls and 50 dementia patients divided into two groups: those with mild and moderate dementia (MMSE 11–23) and patients suffering from severe dementia (MMSE 0–10). Cognitive functions of the subjects were assessed using the Mini Mental State Examination (MMSE). Enzymatic and non-enzymatic antioxidants, oxidative damage products and protein glycoxidative modifications were determined in non-stimulated (NWS) and stimulated (SWS) saliva as well as erythrocyte/plasma samples. Generally, in dementia patients, we observed the depletion of antioxidant defences leading to oxidative and glycoxidative damage in NWS, SWS and blood samples. Both salivary and blood oxidative stress increased with the severity of the disease, and correlated with a decrease of cognitive functions. Interestingly, in dementia patients, reduced glutathione (GSH) in NWS correlated not only with the severity of dementia, but also with GSH concentration in the plasma. In receiver operating characteristic (ROC) analysis, we have demonstrated that salivary GSH clearly distinguishes patients with severe dementia from those suffering from mild or moderate dementia (area under the curve (AUC) = 1). Therefore, salivary GSH can be used as a non-invasive biomarker of cognitive impairment.

## 1. Introduction

Dementia is a syndrome of gradual deterioration of cognitive functions, accompanied by behavioural disorders and difficulties in everyday functioning. According the World Health Organization (WHO), nowadays this problem affects about 1% of the general population (mainly woman), and as many as 7.7 million new patients become affected every year [1,2]. Numerous epidemiological studies have demonstrated that the most common cause of dementia is Alzheimer’s disease (AD) [3]. AD is a degenerative brain disease caused by the accumulation of pathological proteins (amyloid β, Tau protein and α-synuclein) in this organ, resulting in the loss of neurons and the connections between them [4]. Although different types of dementia have different clinical pictures, it is believed that they are based on similar mechanisms, leading to neurodegeneration. This state is undoubtedly related to oxidative stress (OS) caused by the imbalance between the formation of reactive oxygen species (ROS) as well as reactive nitrogen species (RNS) and the antioxidant balance of the body [4,5]. Excess free radicals damage cellular components (i.e., proteins, lipids and DNA), but the oxidation of enzymatic and signalling proteins is particularly dangerous for the body. It has been shown that the accumulation of protein oxidation products plays a key role in the pathogenesis of neurodegenerative diseases [6,7,8]. Importantly, increased concentrations of protein oxidation products are found not only in the brain and cerebrospinal fluid, but also in peripheral tissues and bioliquids [7,9,10]. Therefore, protein oxidation products are more and more often used in laboratory diagnostics in psychiatry [7,10,11].

As demonstrated in our earlier studies, saliva may be an alternative laboratory material used in the diagnosis of a moderate stage of dementia [12]. Indeed, unlike blood and cerebrospinal fluid, saliva is an easily accessible, non-infectious, cheap bioliquid collected in a non-invasive manner, in which the concentration of most compounds reflects their content in blood and tissue [13,14]. Saliva is produced by the large salivary glands: sublingual, parotid and submandibular glands as well as numerous smaller salivary glands situated at the bottom of the oral cavity. Important components of saliva are antioxidant molecules, which is not surprising as the oral cavity is the first line of defence against oxygen free radicals [15]. In patients with dementia, the antioxidant properties of saliva are reduced, the level of oxidative products of DNA, protein and lipid damage is increased, which is accompanied by reduction of saliva secretion [12,16]. As demonstrated earlier, salivary AGEs (advanced glycation end products) may be one of the non-invasive biomarkers used in the diagnosis of moderate dementia [10,12]. However, there are still no indicators to assess the severity of dementia. Therefore, the aim of this study was to evaluate the salivary redox balance and protein oxidation products in saliva as potential non-invasive biomarkers differentiating moderate from severe stages of dementia. It is also advisable to examine the correlations between these parameters in saliva and plasma/erythrocytes in patients with dementia. Still little is known about the changes in redox homeostasis and protein oxidative damage in patients with different types or stages of dementia.

## 2. Materials and Methods

### 2.1. Ethical Issues

The study was approved by the Bioethics Committee of the Medical University of Bialystok, Poland (permission number R-I-002/62/2016). After a detailed explanation of the purpose of our research and the possible risk, all patients agreed in writing to participate in the experiment.

### 2.2. Patients

The study included 50 patients with dementia (35 women and 15 men; mean age 80.24 ± 6.42) treated from January 2017 to July 2017 at the Psychogeriatrics Department of the Stanisław Deresz Independent Public Psychiatric Healthcare Clinic in Choroszcz, Poland. Based on psychiatric, psychological and additional examinations, Alzheimer type of dementia (15 people), vascular dementia (19 people) and mixed dementia (16 people) were diagnosed in the patients. Cognitive functions were assessed using the Mini Mental State Examination (MMSE). The study participants were divided into two groups: those with mild and moderate dementia (MMSE 11–23) and patients with severe dementia (MMSE 0–10). One experienced psychiatrist (N. W.) qualified all the patients for the examinations.

The control group, similar to the study group in terms of gender and age, consisted of 50 patients reporting for follow-up appointments to the Department of Restorative Dentistry of the Medical University of Bialystok from March 2017 to September 2017.

The criteria for inclusion in and exclusion from the study are presented in Table 1.

The number of patients was set based on a previously conducted pilot study. The power of the study was set at 0.8.

### 2.3. Research Material

The research material consisted of venous blood, non-stimulated whole saliva (NWS) and stimulated whole saliva (SWS) collected from patients via the spitting method. Material for all assays was collected prior to pharmacological and psychiatric treatment of the patients.

### 2.4. Blood Collection

Fasting venous blood (10 mL) was collected after an overnight rest period using S-Monovette^®^ K3 EDTA blood collection system (Sarstedt). After blood collection, the samples were centrifuged at 1500 *g* (10 min, +4 °C; MPW 351, MPW Med. Instruments, Warsaw, Poland). No haemolysis was observed in any of the samples. Plasma forming the upper layer was collected, and erythrocytes were rinsed three times with a cold solution of 0.9% NaCl and haemolysed by adding 9 volumes of cold 50 mM phosphate buffer with pH 7.4 (1:9, *v*/*v*) [17]. Butylated hydroxytoluene (BHT) antioxidant was added to the samples to protect them against oxidation (5 μL 0.5 M BHT in acetonitrile per 0.5 mL plasma/erythrocytes) [18]. The samples were stored at −80 °C (but not longer than half a year) until assayed.

### 2.5. Saliva Collection

For at least 2 h before saliva collection, the patients refrained from eating and drinking (except for clean water) and did not perform any oral hygiene procedures. Moreover, at least 8 h prior to saliva collection, participants from the study/control group did not take any medications. Saliva was collected [19,20]:

- Between 8 am and 10 am (in order to minimize the effect of daily rhythms on saliva secretion) in a room providing intimate conditions,
- After rinsing the oral cavity three times with distilled water at room temperature,
- In a seated position, with the head slightly inclined downwards, and minimised facial and lip movements,
- The saliva accumulated at the bottom of the oral cavity was spat out into a sterile Falcon tube placed in an ice container. The saliva collected within the first minute was disposed of.

NWS was collected for 10 min. After a 5 min interval, SWS was taken by sprinkling 10 µL of 2% citric acid on the tip of the tongue every 30 s. SWS was collected for 5 min to a maximum volume of 5 mL [19,20]. When collected, the saliva was immediately centrifuged (3000 *g*, 20 min, +4 °C). BHT (5 μL 0.5 M BHT in acetonitrile per 0.5 mL supernatant saliva) was added to the obtained supernatants to protect them against oxidation, and then frozen at −80 °C (but not for longer than 6 months) [18].

### 2.6. Dental Examination

Dental examination was conducted in accordance with the World Health Organization criteria [21] in artificial lighting, using a mirror, an explorer and a periodontal probe. All the examinations were performed by the same dentist (A.K.) immediately after the saliva collection. DMFT (decay, missing, filled teeth), PBI (Papilla Bleeding Index), GI (Gingival Index) and the occurrence of carious lesions of root cement (CR) were determined. The DMFT index is the sum of teeth with caries (D), teeth extracted because of caries (M) and teeth filled because of caries (F). The PBI showed the intensity of bleeding from the gingival papilla after probing. GI criteria include qualitative changes in the gingiva [21]. Thirty patients had the interrater agreements assessed. The reliability for DMFT was *r* = 0.97; for PBI: *r* = 0.96; and for GI: *r* = 0.99.

### 2.7. Redox Assays

Antioxidant defence (salivary peroxidase (SP), glutathione peroxidase (GPx), catalase (CAT), superoxide dismutase (SOD), reduced glutathione (GSH), total antioxidant status (TAS)), protein oxidative damage products (advanced oxidation protein products (AOPP), Amadori products, advanced glycation end products (AGE), protein carbonyls (PC), total thiols) and protein glycoxidative modifications (dityrosine, kynurenine, N-formylkynurenine and tryptophan) were determined in non-stimulated (NWS) and stimulated (SWS) saliva as well as in the erythrocyte/plasma samples of patients from the study and the control group. The absorbance/fluorescence was measured using Infinite M200 PRO Multimode Microplate Reader, Tecan. All determinations were performed in duplicate samples and standardized to 1 mg of total protein. All reagents (unless stated otherwise) were obtained from Sigma-Aldrich, Nümbrecht, Germany and/or Sigma-Aldrich, Saint Louis, MO, USA.

### 2.8. Antioxidant Assays

SP activity was analysed spectrophotometrically at 412 nm based on the reduction of 5,5′-dithio-bis-(2-nitrobenzoic acid) (DTNB) to thionitrobenzene acid [22]. A decrease in the absorbance of thionitrobenzene acid was measured five times at 30-s intervals. GPx activity was determined spectrophotometrically at 340 nm based on the reduction of organic peroxides in the presence of NADPH [23]. It was assumed that one unit of GPx catalyses the process of oxidation of 1 μmol NADPH per one minute. CAT activity was assayed spectrophotometrically at 240 nm by measuring the rate of hydrogen peroxide decomposition [24]. One unit of CAT activity was defined as the amount of the enzyme that decomposes 1 mmol hydrogen peroxide per one minute. SOD activity was determined spectrophotometrically at 480 nm by measuring the inhibition rate of adrenaline oxidation to adrenochrome [25]. It was assumed that one unit of SOD activity inhibits the oxidation of adrenaline by 50%. The concentration of GSH was estimated colorimetrically using Ellman’s method with DTNB [26]. The absorbance was measured at 412 nm. TAS was determined using a commercial kit (Randox Total Antioxidant Status (TAS), Crumlin, UK) according to the manufacturer’s instructions. This assay involved the conversion of metmyoglobin into ferrylmyoglobin in the presence of iron ions. The absorbance was measured at 600 nm.

### 2.9. Oxidative Damage Assays

AOPP concentration was estimated spectrophotometrically at 340 nm by measuring the oxidative capacity of iodine ion [27]. AGE content was analysed spectrofluorimetrically by measuring AGE-specific fluorescence at 350 nm/440 nm [27]. For AOPP and AGE determination, plasma samples were diluted 1:50 (*v*/*v*) in phosphate buffered saline (PBS) [17]. The formation of Amadori products was determined colorimetrically using Nitro Blue Tetrazolium (NBT) assay [28]. The absorbance was measured at 525 nm with the use of extinction coefficient for monoformazan (12,640 M^−1^ cm^−1^). The concentration of PC was determined colorimetrically based on the reaction of 2,4-dinitrophenylhydrazine (2,4-DNPH) with carbonyl groups in the oxidatively damaged proteins [29]. The absorbance was measured at 355 nm and absorption coefficient for 2,4-DNPH (22,000 M^−1^ cm^−1^) was used. The total thiol levels were measured colorimetrically using the Ellman’s reagent (DTNB) in 0.1 M phosphate buffer, pH 8.0 [30]. The absorbance was measured at 412 nm, and thiol group content was calculated on the basis of a standard curve with GSH as a standard.

### 2.10. Protein Glycoxidation Assays

To detect dityrosine, kynurenine, N-formylkynurenine and tryptophan, saliva and plasma samples were diluted in 0.1 M H_2_SO_4_ (1:5, *v*/*v*), and the characteristic fluorescence at 330/415, 365/480, 325/434 and 95/340 nm, respectively, was measured [31]. The results were normalized to the fluorescence of 0.1 mg/mL quinine sulphate in 0.1 M H_2_SO_4_.

### 2.11. Total Protein Assay

The concentration of total protein was determined using the commercial kit Thermo Scientific PIERCE BCA Protein Assay (No. 23225, Rockfold, IL, USA) with bovine serum albumin (BSA) as a standard.

### 2.12. Statistical Analysis

Statistical analysis was performed using GraphPad Prism 7 for Mac (GraphPad Software, La Jolla, USA). The results were expressed as mean ±SEM. One-way ANOVA, Bonferroni’s multiple comparisons test, Student’s *t*-test and Pearson’s correlation method were used. The area under the curve (AUC) and confidence intervals were determined based on receiver operating characteristic (ROC). Multiplicity adjusted *p* value was also calculated. The statistical significance was defined as *p* ≤ 0.05.

## 3. Results

### 3.1. Clinical Characteristics

Clinical data of dementia patients and the control group are presented in Table 2. No significant differences in either biochemical parameters or blood morphology were found between the groups. Only cognitive functions in the Mini Mental State Examination (MMSE) and formal education in years were statistically different between patients with dementia as compared to the controls. 

### 3.2. Dental Examination

The flow of non-stimulated and stimulated saliva as well as total protein concentration in NWS and SWS were significantly lower in patients with moderate and mild dementia (11–23 MMSE) and severe dementia (0–10 MMSE) compared to the controls. However, no significant differences in the condition of the dentition and periodontium were found in patients with dementia compared to the control group (Table 3).

### 3.3. Antioxidant Defences

**Figure 1:**
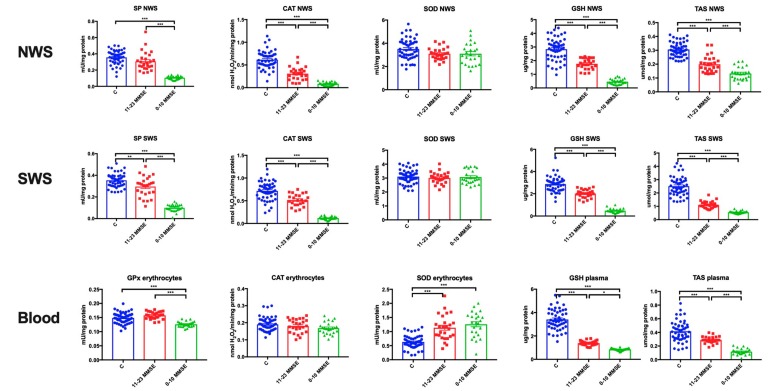
Antioxidant defence and redox homeostasis in the non-stimulated and stimulated saliva as well as erythrocytes and plasma of patients in different stages of dementia, and healthy controls. Abbreviations: C—the control; CAT—catalase; GPx—glutathione peroxidase; GSH—reduced glutathione; MMSE—Mini Mental State Examination; NWS—non-stimulated whole saliva; SP—salivary peroxidase; SOD—superoxide dismutase; SWS—stimulated whole saliva; TAS—total antioxidant status. * <0.05, ** <0.01, *** <0.001.

The activity of CAT (*p* < 0.001 in both groups) as well as the level of GSH (*p* < 0.001 in both groups) and TAS (*p* < 0.001 in both groups) were significantly lower in NWS in patients with mild and moderate as well as severe dementia compared to the control group. The activity of SP was considerably lower only in NWS of subjects with severe dementia compared to the controls (*p* < 0.001). The activity of SP and CAT as well as the level of GSH and TAS were significantly lower in NWS of severe dementia patients compared to those with mild and moderate dementia (*p* < 0.001 in all groups) (Figure 1).

In SWS, the activity of SP (*p* < 0.01, *p* < 0. 001, respectively) and CAT (*p* < 0.001 in both groups), the level of GSH (*p* < 0.001 in both groups) and TAS (*p* < 0.001 in both groups) was significantly lower in patients with mild and moderate as well as severe dementia compared to the control group. In SWS, the activity of SP and CAT, and the level of GSH and TAS were considerably lower in patients with severe dementia compared to those with mild and moderate dementia (*p* < 0.001 in all groups) (Figure 1).

The concentration of GSH (*p* < 0.001 in both groups) and TAS (*p* < 0.001 in both groups) in plasma were significantly lower in patients with mild and moderate as well as severe dementia compared to the control group. The activity of GPx was considerably lower only in erythrocytes of subjects with severe dementia compared to the controls (*p* < 0.001). However, the activity of SOD (*p* < 0.001 in both groups) in erythrocytes of patients with mild, moderate and severe dementia was considerably higher in comparison with the controls. Within the study group, GPx activity in erythrocytes, as well as GSH and TAS concentrations in plasma were significantly lower in patients with severe dementia compared to those with mild and moderate dementia (*p* < 0.001, *p* < 0.05, *p* < 0.001, respectively) (Figure 1). 

### 3.4. Oxidative Damage to Proteins

**Figure 2:**
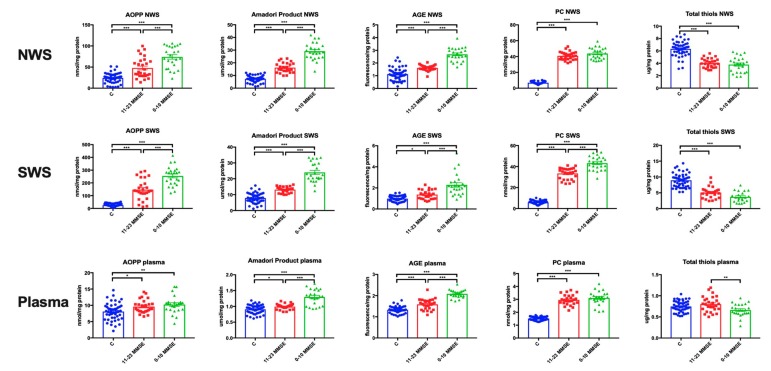
Oxidative damage in non-stimulated and stimulated saliva as well as plasma of patients in different stages of dementia, and healthy controls. Abbreviations: AGE—advanced glycation end products; AOPP—advanced oxidation protein products; C—the control; MMSE—Mini Mental State Examination; NWS—non-stimulated whole saliva; PC—protein carbonyls; SWS—stimulated whole saliva. * <0.05, ** <0.01, *** <0.001.

In NWS, the concentrations of AOPP (*p* < 0.001 in both groups), Amadori products (*p* < 0.001 in both groups), PC (*p* < 0.001 in both groups) and AGE fluorescence (*p* < 0.001 in both groups) were significantly higher in patients with mild, moderate and severe dementia compared to the control group. The total thiol concentration (*p* < 0.001 in both groups) in NWS was considerably lower in patients with any stage of dementia compared to the controls. In NWS, the concentration of AOPP (*p* < 0.001), Amadori product (*p* < 0.001) and AGE fluorescence (*p* < 0.001) in severe dementia patients was significantly higher than in the subjects with mild or moderate dementia (Figure 2).

In SWS, the concentration of AOPP (*p* < 0.001 in both groups), Amadori products (*p* < 0.001 in both groups), PC (*p* < 0.001 in both groups) and AGE fluorescence (*p* < 0.05, *p* < 0.001, respectively) were significantly higher in patients with mild and moderate dementia as well as severe dementia compared to the control group. Total thiol concentration (*p* < 0.001 in both groups) was considerably lower in SWS of patients with mild, moderate and severe dementia compared to the controls. In SWS, the concentration of AOPP (*p* < 0.001), Amadori products (*p* < 0.001), PC (*p* < 0.001) and AGE fluorescence (*p* < 0.001) in patients with severe dementia was significantly higher than in patients with mild and moderate dementia (Figure 2).

In plasma, the concentration of AOPP (*p* < 0.05, *p* < 0.01, respectively), Amadori products (*p* < 0.05, *p* < 0.001, respectively), PC (*p* < 0.001 in both groups) and AGE fluorescence (*p* < 0.001 in both cases) were significantly higher in patients with mild and moderate as well as severe dementia compared to the control group. The content of Amadori products (*p* < 0.001) and AGE fluorescence (*p* < 0.001) in plasma of patients with severe dementia were considerably higher compared to patients with mild and moderate dementia, while the concentration of total thiols (*p* < 0.01) was significantly lower (Figure 2).

### 3.5. Protein Glycoxidation

**Figure 3:**
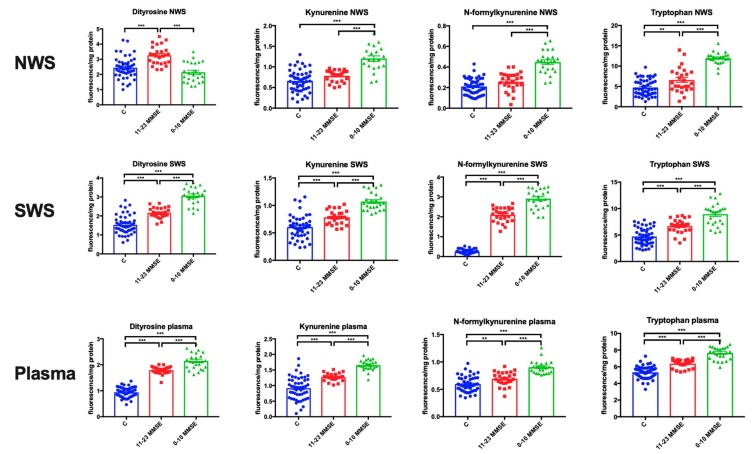
Protein glycoxidation in non-stimulated and stimulated saliva as well as plasma of patients in different stages of dementia, and healthy controls. Abbreviations: C—the control; MMSE—Mini Mental State Examination; NWS—non-stimulated whole saliva; SWS—stimulated whole saliva. ** <0.01, *** <0.001.

In NWS, tryptophan fluorescence (*p* < 0.01, *p* < 0.001, respectively) was significantly higher in subjects with mild and moderate as well as severe dementia compared to the control group, while the fluorescence of kynurenine (*p* < 0.001) and N-formylkynurenine (*p* < 0.001) was significantly higher only in subjects with severe dementia compared to the control group. Dityrosine fluorescence (*p* < 0.001) in NWS of patients with mild and moderate dementia was considerably higher in comparison with the control group. The mean value of kynurenine (*p* < 0.001), N-formylkynurenine (*p* < 0.001) and tryptophan (*p* < 0.001) fluorescence in NWS of patients with severe dementia was significantly higher compared to patients with mild and moderate dementia, while dityrosine fluorescence (*p* < 0.001) was considerably lower in patients with severe dementia (Figure 3).

In SWS, the fluorescence of dityrosine (*p* < 0.001 in both groups), kynurenine (*p* < 0.001 in both groups), N-formylkynurenine (*p* < 0.001 in both groups) and tryptophan (*p* < 0.001 in both groups) in patients with mild and moderate as well as severe dementia was significantly higher than in the control group. The content of dityrosine, kynurenine, N-formylkynurenine and tryptophan in SWS of patients with severe dementia was largely higher in comparison with the patients with mild and moderate dementia (*p* < 0.001 in all groups) (Figure 3).

In plasma, the fluorescence of dityrosine (*p* < 0.001 in both groups), kynurenine (*p* < 0.001 in both groups), N-formylkynurenine (*p* < 0.01, *p* < 0.001, respectively) and tryptophan (*p* < 0.001 in both groups) in patients with mild to moderate as well as severe dementia was significantly higher compared to the control group. The content of dityrosine, kynurenine, N-formylkynurenine and tryptophan in plasma of patients with severe dementia was significantly higher compared to those with mild and moderate dementia (*p* < 0.001 in all groups) (Figure 3)

### 3.6. Effects of the Type of Dementia, Gender and Age of Patients

No differences were found in the assayed redox biomarkers of patients with Alzheimer’s dementia, vascular dementia and mixed dementia. Similarly, we observed no influence of sex and age of the patients on the assessed parameters of oxidative stress.

### 3.7. Correlations

**Figure 4:**
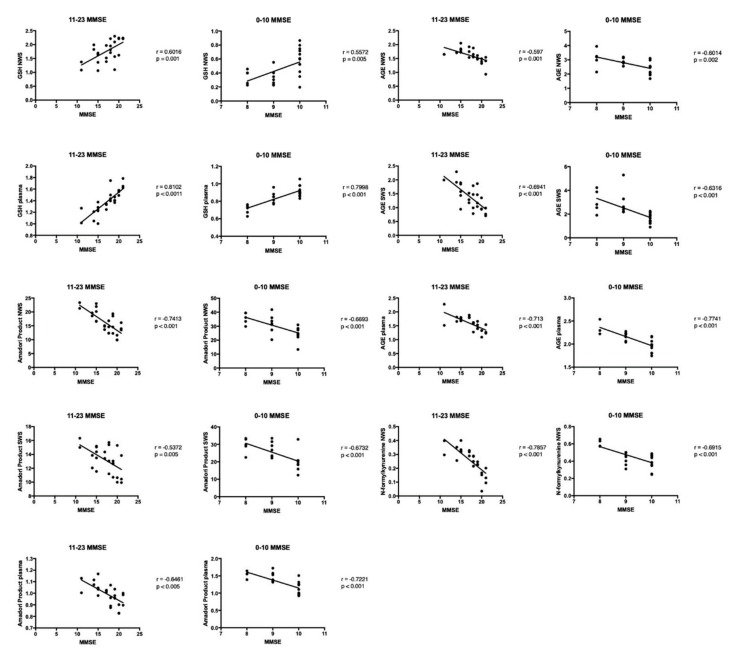
Correlations between oxidative stress biomarkers and clinical status in dementia patients. Abbreviations: AGE—advanced glycation end products; GSH—reduced glutathione; MMSE—Mini Mental State Examination; NWS—non-stimulated whole saliva; SWS—stimulated whole saliva.

**Figure 5:**
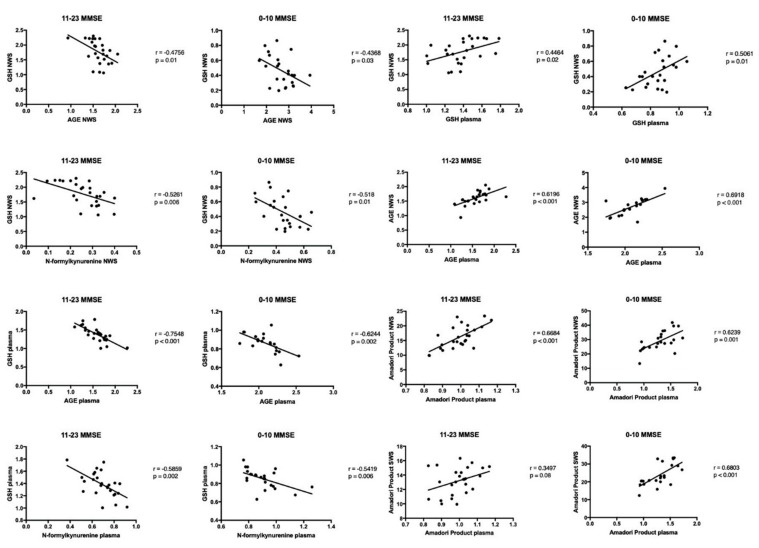
Correlations between oxidative stress biomarkers in dementia patients. Abbreviations: AGE—advanced glycation end products; GSH—reduced glutathione; MMSE—Mini Mental State Examination; NWS—non-stimulated whole saliva; SWS—stimulated whole saliva.

The results of statistically significant correlations are presented in Figure 4 and Figure 5. Interestingly, in the group of patients with dementia, the concentration of GSH in NWS and blood plasma correlated positively with the decrease of cognitive functions in the MMSE scale. The content of Amadori products and AGE in NWS, SWS and blood plasma correlated negatively with the degree of dementia progression in the MMSE scale. Similar correlations were observed for N-formylkynurenine in non-stimulated saliva of dementia patients. The concentration of GSH correlated negatively with N-formylkynurenine fluorescence in non-stimulated saliva of dementia patients. There was a negative correlation between GSH concentration and AGE content in the plasma of dementia patients. Similarly, we observed a negative correlation between plasma GSH concentration and fluorescence of N-formylkynurenine. A positive correlation between GSH concentration in NWS and GSH concentration in blood plasma was also found in the group of patients with dementia. Moreover, a positive correlation was found between AGE content in NWS and plasma, and between the concentration of Amadori products in NWS, SWS and plasma.

### 3.8. ROC Analysis

**Figure 6:**
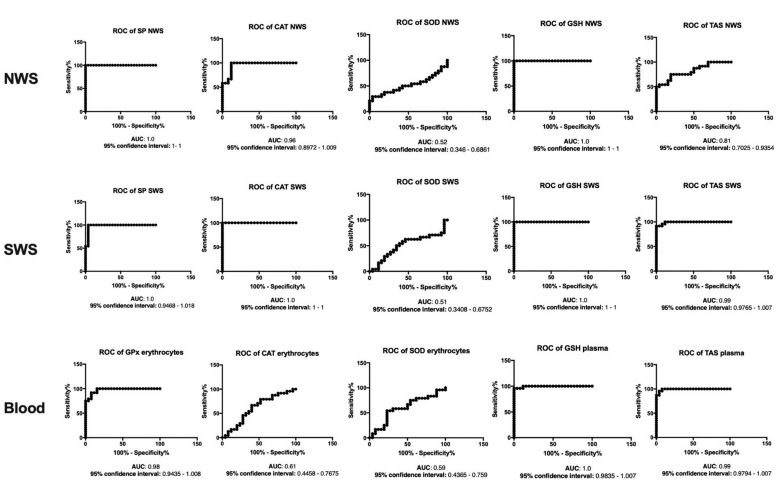
Receiver operating characteristic (ROC) analysis of antioxidants in non-stimulated and stimulated saliva as well as plasma of patients in different stages of dementia. Abbreviations: AUC—area under the curve; CAT—catalase; GPx—glutathione peroxidase; GSH—reduced glutathione; NWS—non-stimulated whole saliva; SP—salivary peroxidase; SOD—superoxide dismutase; SWS—stimulated whole saliva; TAS—total antioxidant status.

**Figure 7:**
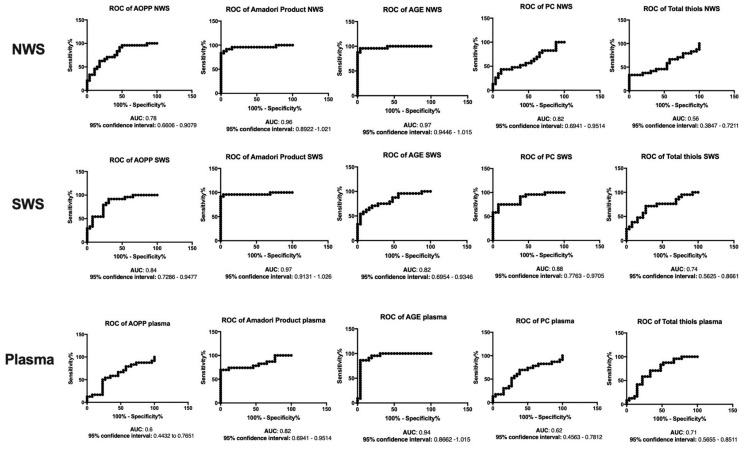
Receiver operating characteristic (ROC) analysis of oxidative damage products in non-stimulated and stimulated saliva as well as plasma of patients in different stages of dementia. Abbreviations: AGE—advanced glycation end products; AOPP—advanced oxidation protein products; AUC—area under the curve; NWS—non-stimulated whole saliva; PC—protein carbonyls; SWS—stimulated whole saliva.

**Figure 8:**
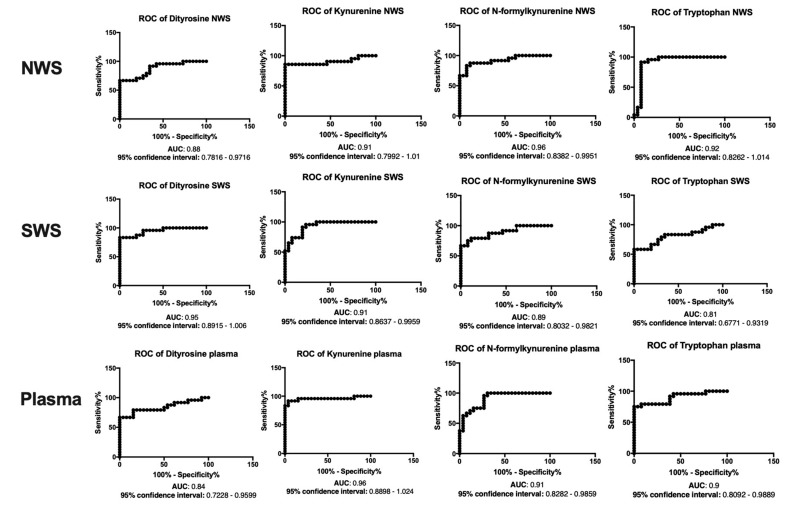
Receiver operating characteristic (ROC) analysis of protein glycoxidation products in non-stimulated and stimulated saliva as well as plasma of patients in different stages of dementia. Abbreviations: AUC—area under the curve; NWS—non-stimulated whole saliva; PC—protein carbonyls; SWS—stimulated whole saliva.

The results of ROC analysis for oxidative stress parameters are presented in Figure 6, Figure 7 and Figure 8. Interestingly, the evaluation of SP/GPx activity, as well as GSH and Amadori products concentration in NWS, SWS and blood showed a very high diagnostic usefulness in differentiating patients with severe dementia from those suffering from mild and moderate dementia. 

## 4. Discussion

This study is the first to evaluate both the antioxidant barrier and oxidative damage to proteins in saliva/blood of patients with varying degrees of dementia progression. We observed the depletion of antioxidant reserves in elderly people with dementia, leading to oxidative and glycoxidative damage to saliva and plasma/erythrocyte proteins. Interestingly, both salivary and central oxidative stress increases with the severity of the disease and correlates with a decrease in cognitive functions. Therefore, selected redox biomarkers may be helpful in the differential diagnosis of dementia.

Numerous epidemiological studies confirm the contribution of oxidative stress to the pathogenesis of the majority of modern diseases [7,16,32,33]. The occurrence of oxidoreductive imbalance has been demonstrated in metabolic diseases, diseases with impaired body defence mechanisms (infectious, inflammatory and autoimmune diseases) and those causing cell proliferation disorders (cancer and genetic diseases) and damage to the central nervous system (neurodegenerative diseases) [32,33]. It is therefore not surprising that redox status biomarkers are more and more frequently used in clinical laboratory diagnostics [10,34]. Although direct analysis of ROS concentration is very difficult, oxidative stress is assessed using mainly antioxidants as well as compounds formed as a result of reactions of free radicals with components of body cells.

The central nervous system is particularly sensitive to redox imbalance compared to other body systems [35,36]. This is due to increased oxygen metabolism of the brain, high content of unsaturated fatty acids and relatively low activity of neuronal antioxidant enzymes. In addition, the exceptionally high content of transition metal ions in brain tissue increases the production of free radicals, which not only damages neurons, but also leads to the autoxidation of neurotransmitters (e.g., dopamine, serotonin and noradrenaline) and impairment of their biological functions [35,36]. In order to counteract the harmful effects of oxidative stress, living organisms have developed enzymatic and non-enzymatic antioxidant mechanisms aimed at preventing the formation of ROS as well as interrupting free radical reactions and inhibiting the interaction of ROS with cellular components [32].

In the group of dementia patients, we found a significant weakening of the antioxidant barrier expressed as a decrease in the activity of antioxidant enzymes (↓SP, ↓CAT) and in the concentration of GSH and TAS in both non-stimulated and stimulated saliva as well as plasma. Interestingly, we also observed a considerable decrease in GSH level in patients with severe dementia compared to those with mild to moderate dementia (in NWS, SWS and plasma). A similar trend was observed for total antioxidant status (TAS) that describes the resultant capacity of all antioxidant systems of the body (i.e., enzymatic and non-enzymatic oxygen free radical scavengers) [34]. Thus, the antioxidative defence of saliva/blood in dementia patients is visibly impaired and increases along the occurrence of cognitive impairment.

It is believed that the most important brain antioxidant is glutathione, i.e., gamma-glutamylcysteinylglycine (GSH) [37]. Due to the presence of thiol groups (-SH), this compound maintains the physiological redox balance of the cell. Additionally, GSH participates in the growth, differentiation, proliferation and apoptosis as well as the reconstruction of oxidatively damaged cellular components, mainly proteins and lipids of cell membranes. A decrease of intracellular GSH concentration entails the intensification of oxidative damage to the brain, and is considered one of the most important causes of neurodegenerative diseases [37,38]. Studies involving the use of nuclear magnetic resonance spectroscopy (NMR) have demonstrated reduced levels of GSH in the brain of patients with AD and other neurological disorders [37,39]. Moreover, it has been observed that the level of GSH is reduced not only in the course of AD, but also in mild cognitive impairment (MCI) considered a preclinical stage of AD [40]. Thus, disturbances in glutathione metabolism precede the occurrence of AD and may be used as early biomarkers of dementia. In our study, GSH levels in NWS, SWS and plasma were significantly lower in patients with severe dementia compared to those with moderate dementia and healthy controls. This fact is not surprising, as in oxidative stress conditions, our antioxidative reserves (mainly -SH groups of enzymatic protein and glutathione) are consumed at a rate proportional to the intensity of ROS production [41,42]. Interestingly, salivary GSH concentration in NWS correlated not only with the severity of dementia, but also with GSH concentration in blood plasma. This indicates the potential use of saliva as a diagnostic material alternative to blood. It is worth noting that saliva collection is non-invasive, painless and very convenient for the elderly as it does not require the involvement of specialised medical staff [14,18]. The high diagnostic usefulness of salivary GSH in the diagnosis of dementia is also confirmed by the results of the ROC analysis. GSH measured in non-stimulated and/or stimulated saliva clearly distinguishes patients with severe dementia from those suffering from mild or moderate dementia (AUC = 1; sensitivity = 100%; specificity = 100%) and can therefore be used as a non-invasive biomarker of cognitive impairment.

Disturbances of redox homeostasis in favour of oxidation processes result in oxidative damage to proteins, lipids and nucleic acids. However, it is proteins, as the main components of the cell, that are the primary target of the attack of oxygen free radicals [7,9]. It is believed that oxidative damage to proteins (i.e., oxidation of thiol groups, nitration of aromatic amino acid and breaking of polypeptide chains with simultaneous formation of cross-links) may determine the exposure to ROS and act as a biomarker of oxidative stress intensity [7,9,32]. Protein oxidation products are much more durable than oxygen free radicals, and also provide information on the consequences of oxidative damage to the body [6,19].

We have demonstrated severe oxidative damage to proteins (↑AOPP, ↑Amadori products, ↑AGE, ↑PC, ↑protein glycoxidative modifications, ↓total thiols) in NWS, SWS and plasma of patients with dementia compared to the controls. Importantly, the degree of protein damage increased along with cognitive function impairment in patients with various types of dementia. It is not surprising because oxidized proteins tend to form aggregates, inhibit the enzymes that break them down and accumulate in metabolically active tissues (e.g., liver, kidney, even the brain) [43,44,45]. However, aromatic amino acids are extremely susceptible to oxidation [8,31], as confirmed by the results of our study: we observed increased content of protein glycoxidative products (↑AGE, ↑dityrosine, ↑kynurenine and ↑N-formylkynurenine) both in saliva and plasma of patients with dementia.

Oxidatively/glycoxidatively modified proteins demonstrate a loss or decrease in biological activity [44]. However, oxidized aromatic amino acid residues (particularly tyrosine, i.e., dityrosine, kynurenine and N-formylkynurenine) may also be cytotoxic [7]. Interestingly, the ability of oxidized proteins to aggregate and accumulate is the basis for ageing and neuronal degeneration processes. The deposition of advanced oxidation products (AOPP) and advanced glycation end products (AGE) of proteins in the brain is particularly dangerous because—when bound to a specific receptor (RAGE; receptor for advanced glycation end products)—these compounds intensify the activity of NADPH (NOX) oxidase, which is the main source of ROS in the cell [36,46,47]. Under the influence of AOPP and AGE, in addition to ROS overproduction, the production of inflammatory mediators is also increased, which further intensifies the formation of free radicals in the positive feedback mechanism (ROS-induced ROS release) [46,47]. Indeed, ROS induce mitogen-activated protein kinase (MAP kinase) and nuclear transcription factor (NF-κB), which enhances the expression of proinflammatory cytokines, chemokines and adhesion molecules as well as boosts the production of oxygen and nitrogen free radicals [48,49]. Importantly, increased formation of AOPP and AGE occurs particularly in the case of glutathione deficiency, as confirmed in our study by the positive correlation observed between GSH and AGE concentrations in plasma of patients with dementia [37,47]. The precursors to the production of AGE are Amadori products (early glycation products), i.e., stable chemical compounds with free carbonyl groups, which—in the presence of oxygen—undergo the processes of oxidation, condensation and crosslinking [9,43]. The concentration of Amadori products in non-stimulated saliva correlated with their content in blood plasma as well as with cognitive impairment in the MMSE scale. This indicates the possibility of using this biomarker in psychiatric laboratory medicine. The results of ROC analysis also have proven high usefulness of Amadori products in differentiating severe dementia from its mild and moderate stages.

Although individual types of dementia are characterised by varied etiology, we did not observe significant differences in the redox biomarkers used in patients with different types of dementia (Alzheimer’s dementia, vascular dementia and mixed dementia). Thus, it can be assumed that dementia is caused by similar disturbances of antioxidant systems as well as oxidative stress [12]. It is suggested that actions reducing the rate of ROS production and/or preventing oxidative damage and accumulation of oxidized proteins may be one of the therapeutic strategies to prevent and eliminate dementia.

Modern medicine does not offer a simple, inexpensive and non-invasive method for early diagnosis of dementia, preferably to be performed before the occurrence of any clinical symptoms [1]. The hope for such screening tests may be salivary redox diagnostics to complement psychiatric and psychological tests. However, it should be recalled that these biomarkers are not exclusively specific to dementia. Oxidative stress is associated with many neurodegenerative and general diseases [7,16,32,33], nevertheless, salivary antioxidants and oxidative damage products are more and more frequently used as diagnostic biomarkers [10,13,20]. Bearing in mind that still little is known about the role of oxidative stress in dementia and neurodegeneration, we would like to highlight the need for further research to assess the diagnostic usefulness of redox biomarkers on a large population of dementia patients. 

Despite the undoubted advantages, our work also had certain limitations. The main one was a relatively small number of patients and the need to include patients with hypertension and ischemic heart disease that very often affect people in this age. However, our study had strictly outlined inclusion and exclusion criteria, particularly with regard to other concomitant diseases and medication taken. Additionally, it was the first study comparing the selected oxidative stress biomarkers in patients with mild and moderate dementia as well as severe dementia at both central (plasma, erythrocytes) and local (saliva) level.

## 5. Conclusions

To sum up, we have demonstrated a significant decrease in antioxidative defence and increased oxidative damage to proteins in non-stimulated and stimulated saliva as well as in erythrocytes/plasma of patients with severe dementia compared to mild and moderate dementia as well as age- and gender-matched healthy controls. Importantly, oxidative stress increases in the course of dementia with the decrease of cognitive function. Since similar redox imbalances and oxidative stress are the cause of individual types of dementia, it is suggested that antioxidant supplementation may reduce the severity of dementia. Furthermore, we have demonstrated that oxidant/antioxidant status disorders occur not only at the central, but also at the local level, which makes saliva a reliable biological material to be used for the diagnosis/monitoring of dementia. However, further studies are needed to confirm the diagnostic usefulness of salivary redox biomarkers in a larger population of patients.

## Figures and Tables

**Table 1:** Inclusion and exclusion criteria for dementia patients and the control group. Abbreviations: ALT—alanine transferase; ASPAT—aspartate transaminase; BMI—body mass index; CRP—C-reactive protein; MMSE—mini mental state examination; TSH—thyroid-stimulating hormone.

	Inclusion Criteria	Exclusion Criteria
Control and Study group	- written informed consent to participate in the study - BMI 18.5–24.5 - normal results of blood morphological tests (erythrocytes, leukocytes, haemoglobin, platelets, haematocrit) and biochemical tests (sodium, potassium, creatinine, ASPAT, ALAT, International Normalized Ratio (INR), CRP as well as normal concentration of TSH, calcium, vitamin B12 and folic acid) - no history of psychoactive substance abuse	- presence of chronic systemic diseases (except hypertension, type 2 diabetes, arteriosclerosis and osteoporosis), autoimmune diseases, lung, thyroid, liver, kidney, digestive tract, infectious diseases and immune disorders (HCV and HIV infections) - periodontal diseases - smoking - alcohol misuse disorder - taking any antibiotics, glucocorticoids, dietary supplements and vitamins for the last 3 months - chronic use of non-steroidal anti-inflammatory drugs (NSAIDs)
Study group	- decreased cognitive performance with undisturbed consciousness, visible in the clinical picture and confirmed by the Mini Mental State Examination (MMSE, 30-point scale) in patients with mild to moderate (11–23) and severe (0–10) stage of dementia -at least 6 months of positive history of cognitive disorders	- acute haemorrhagic states and ischaemia, tumours of the central nervous system and normal pressure hydrocephalus in a computed tomography scan of the head
Control group	- no cognitive impairment in the clinical examination - no dementia (MMSE > 27)	- MMSE < 27

**Table 2:** Clinical characteristics of dementia patients and the control group.

Patient Characteristics		Control *n* = 50	11–23 MMSE *n* = 26	0–10 MMSE *n* = 24	ANOVA *p*
Sex	Male *n* (%)	15 (30)	8 (30)	7 (30)	-
	Female *n* (%)	35 (70)	18 (70)	17 (70)	-
Age		80.82 ± 1.15	80.85 ± 1.25	81.17 ± 0.82	0.15
Formal education in years		7.52 ± 0.65	7.81 ± 0.77	5.11 ± 0.58	0.006
MMSE score		27.42 ± 0.52	17.12 ± 0.58	9.28 ± 0.23	˂0.001
Type of dementia	Alzheimer *n* (%)	-	8 (30.77)	7 (29.17)	-
	Vascular *n* (%)	-	11 (42.31)	8 (33.33)	-
	Mixed *n* (%)	-	7 (26.92)	9 (37.5)	-
Ca^2+^ (mEq/L)		4.25 ± 0.11	4.48 ± 0.08	4.42 ± 0.04	0.10
B_12_ (pg/mL)		323.40 ± 82.84	378.10 ± 53.17	483.90 ± 83.40	0.34
Folic acid (ng/mL)		6.23 ± 0.81	5.99 ± 0.89	5.40 ± 0.61	0.71
TSH (uIU/mL)		1.08 ± 0.16	1.39 ± 0.23	1.59 ± 0.22	0.26
CRP		3.16 ± 0.79	3.16 ± 0.98	5.04 ± 1.24	0.34
RBC		4.51 ± 0.14	4.43 ± 0.14	4.47 ± 0.09	0.90
Hb		13.76 ± 0.32	13.55 ± 0.34	13.45 ± 0.27	0.77
Hematocrit		40.88 ± 1.08	40.37 ± 1.08	40.33 ± 0.83	0.91
MCV		91.20 ± 1.02	91.61 ± 1.10	90.46 ± 0.99	0.72
MCH		33.62 ± 0.18	33.60 ± 0.21	33.37 ± 0.16	0.54
RDW		14.90 ± 0.20	14.90 ±0.30	15.14 ± 0.25	0.72
PLT		268.80 ± 15.15	243.70 ± 18.80	260.10 ± 14.23	0.56
MPV		7.95 ± 0.17	8.40 ± 0.22	8.13 ± 0.15	0.23
PCT		0.21 ± 0.01	0.20 ± 0.01	0.21 ± 0.01	0.83
PDW		13.54 ±0.34	13.95 ± 0.36	13.95 ± 0.21	0.54
WBC		7.40 ± 0.39	7.11 ± 0.53	7.25 ± 0.40	0.91
Na^+^		140.90 ± 0.50	141.20 ± 0.48	137.40 ± 4.36	0.61
K^+^		4.20 ± 0.07	4.24 ± 0.08	4.29 ± 0.09	0.74
AST		20.04 ± 1.17	21.30 ± 1.56	24.53 ± 1.73	0.10
ALT		13.79 ± 1,69	17.17 ± 1.81	18.38 ± 1.87	0.19
Glucose		111.50 ± 4.04	111.00 ± 2.77	106.90 ± 4.07	0.62
Creatinine		0.90 ± 0.04	0.81 ± 0.04	0.91 ± 0.05	0.26
Urea		39.79 ± 2.79	39.35 ± 2.46	45.31 ± 2.41	0.17
Hypertension *n* (%)		21 (42)	17 (65.38)	11 (45.83)	-
Diabetes *n* (%)		6 (12)	4 (15.38)	4 (16.67)	-
CHD *n* (%)		9 (18)	7 (26.92)	1 (4.17)	-
Atherosclerosis *n* (%)		6 (12)	3 (11.54)	2 (8.33)	-
Osteoporosis *n* (%)		2 (4)	1 (3.85)	1 (4.17)	-
Medications	<5 drugs/day *n* (%)	27 (54)	12 (46.15)	10 (41.67)	-
	≥5 drugs/day *n* (%)	6 (12)	4 (15.38)	4 (16.67)	-

Abbreviations: ALT—alanine transferase; AST—aspartate transaminase; B_12_—vitamin B_12_; Ca^2+^—calcium; CHD—coronary heart disease; CRP—C-reactive protein; HB—haemoglobin; K^+^—potassium; MCH—mean corpuscular haemoglobin; MCV—mean corpuscular volume; MMSE—mini mental state examination; MPV—mean platelet volume; Na^+^—sodium; NWS—non-stimulated whole saliva; PCT—procalcitonin; PDW—platelet distribution width; PLT—platelets; RBC—red blood cells; RDW—red cell distribution width; SWS—stimulated whole saliva; TSH—thyroid stimulating hormone; WBC—white blood cells.

**Table 3:** Salivary gland function and stomatological characteristics of dementia patients and control subjects.

Patient Characteristics	Control *n* = 50	11–23 MMSE *n* = 26	0–10 MMSE *n* = 24
NWS flow rate (mL/min)	0.5 ± 0.04	0.07 ± 0.01 *	0.06 ± 0.01 *
SWS flow rate (mL/min)	1.34 ± 0.07	0.083 ± 0.01 *	0.07 ± 0.01 *
NWS total protein (µg/mL)	3414 ± 326.1	4806 ± 416.5 *	4537 ± 431.6 *
SWS total protein (µg/mL)	2390 ± 83.57	4449 ± 345.6 *	4718 ± 496.4 *
DMFT	29.32 ± 0.53	29.96 ± 0.89	30.09 ± 0.69
PBI	1.09 ± 0.07	1.52 ± 0.27	2.07 ± 0.24
GI	1.58 ± 0.06	2.06 ± 0.15	2.18 ± 0.14
CR	0.28 ± 0.73	0.13 ± 0.1	0.7 ± 0.48

Abbreviations: CR—root caries; DMFT—decayed, missing, filled teeth index; GI—gingival index; MMSE—Mini Mental State Examination; n—number of patients; NWS—non-stimulated whole saliva; PBI—papilla bleeding index; SWS—stimulated whole saliva. * *p* < 0.05 vs. control group.

## References

[1] Larson E.B., Yaffe K., Langa K.M. (2013). New Insights into the dementia epidemic. N. Engl. J. Med..

[2] Prince M., Comas-Herrera A., Knapp M., Guerchet M., Karagiannidou M. (2016). World Alzheimer Report 2016 Improving Healthcare for People Living with Dementia. Coverage, Quality and Costs Now and In the Future.

[3] Livingston G., Sommerlad A., Orgeta V., Costafreda S.G., Huntley J., Ames D., Ballard C., Banerjee S., Burns A., Cohen-Mansfield J. (2017). Dementia prevention, intervention, and care. Lancet.

[4] Butterfield D.A., Reed T., Newman S.F., Sultana R. (2007). Roles of amyloid β-peptide-associated oxidative stress and brain protein modifications in the pathogenesis of Alzheimer’s disease and mild cognitive impairment. Free Radic. Biol. Med..

[5] Mao P. (2013). Oxidative Stress and its clinical applications in dementia. J. Neurodegener. Dis..

[6] Cobb C.A., Cole M.P. (2015). Oxidative and nitrative stress in neurodegeneration. Neurobiol. Dis..

[7] Dasuri K., Zhang L., Keller J.N. (2013). Oxidative stress, neurodegeneration, and the balance of protein degradation and protein synthesis. Free Radic. Biol. Med..

[8] Robaszkiewicz A., Bartosz G., Soszyński M. (2008). N-chloroamino acids cause oxidative protein modifications in the erythrocyte membrane. Mech. Ageing Dev..

[9] Gonos E.S., Kapetanou M., Sereikaite J., Bartosz G., Naparlo K., Grzesik M., Sadowska-Bartosz I. (2018). Origin and pathophysiology of protein carbonylation, nitration and chlorination in age-related brain diseases and aging. Aging.

[10] Kułak-Bejda A., Waszkiewicz N., Bejda G., Zalewska A., Maciejczyk M. (2019). Diagnostic value of salivary markers in neuropsychiatric disorders. Dis. Markers.

[11] Chang Y.-T., Chang W.-N., Tsai N.-W., Huang C.-C., Kung C.-T., Su Y.-J., Lin W.-C., Cheng B.-C., Su C.-M., Chiang Y.-F. (2014). The roles of biomarkers of oxidative stress and antioxidant in Alzheimer’s disease: A systematic review. Biomed. Res. Int..

[12] Choromańska M., Klimiuk A., Kostecka-Sochoń P., Wilczyńska K., Kwiatkowski M., Okuniewska N., Waszkiewicz N., Zalewska A., Maciejczyk M. (2017). Antioxidant defence, oxidative stress and oxidative damage in saliva, plasma and erythrocytes of dementia patients. Can salivary AGE be a marker of dementia?. Int. J. Mol. Sci..

[13] Javaid M.A., Ahmed A.S., Durand R., Tran S.D. (2016). Saliva as a diagnostic tool for oral and systemic diseases. J. Oral Biol. Craniofacial Res..

[14] Wang J., Schipper H.M., Velly A.M., Mohit S., Gornitsky M. (2015). Salivary biomarkers of oxidative stress: A critical review. Free Radic. Biol. Med..

[15] Żukowski P., Maciejczyk M., Waszkiel D. (2018). Sources of free radicals and oxidative stress in the oral cavity. Arch. Oral Biol..

[16] Tothova L., Kamodyova N., Cervenka T., Celec P. (2015). Salivary markers of oxidative stress in oral diseases. Front. Cell. Infect. Microbiol..

[17] Borys J., Maciejczyk M., Antonowicz B., Krętowski A., Waszkiel D., Bortnik P., Czarniecka-Bargłowska K., Kocisz M., Szulimowska J., Czajkowski M., Waszkiewicz N., Zalewska A. (2018). Exposure to Ti4Al4V titanium alloy leads to redox abnormalities, oxidative stress, and oxidative damage in patients treated for mandible fractures. Oxid. Med. Cell. Longev..

[18] Maciejczyk M., Szulimowska J., Skutnik A., Taranta-Janusz K., Wasilewska A., Wiśniewska N., Zalewska A. (2018). Salivary biomarkers of oxidative stress in children with chronic kidney disease. J. Clin. Med..

[19] Fejfer K., Buczko P., Niczyporuk M., Ładny J.R., Hady H.R., Knaś M., Waszkiel D., Klimiuk A., Zalewska A., Maciejczyk M. (2017). Oxidative modification of biomolecules in the nonstimulated and stimulated saliva of patients with morbid obesity treated with bariatric surgery. Biomed. Res. Int..

[20] Knaś M., Maciejczyk M., Sawicka K., Hady H.R., Niczyporuk M., Ładny J.R., Matczuk J., Waszkiel D., Żendzian-Piotrowska M., Zalewska A. (2016). Impact of morbid obesity and bariatric surgery on antioxidant/oxidant balance of the unstimulated and stimulated human saliva. J. Oral Pathol. Med..

[21] World Health Organization (2013). Oral Health Surveys: Basic Methods.

[22] Mansson-Rahemtulla B., Baldone D.C., Pruitt K.M., Rahemtulla F. (1986). Specific assays for peroxidases in human saliva. Arch. Oral Biol..

[23] Paglia D.E., Valentine W.N. (1967). Studies on the quantitative and qualitative characterization of erythrocyte glutathione peroxidase. J. Lab. Clin. Med..

[24] Aebi H. (1984). Catalase in vitro. Methods in Enzymology.

[25] Misra H.P., Fridovich I. (1972). The role of superoxide anion in the autoxidation of epinephrine and a simple assay for superoxide dismutase. J. Biol. Chem..

[26] Griffith O.W. (1980). Determination of glutathione and glutathione disulfide using glutathione reductase and 2-vinylpyridine. Anal. Biochem..

[27] Kalousová M., Skrha J., Zima T. (2002). Advanced glycation end-products and advanced oxidation protein products in patients with diabetes mellitus. Physiol. Res..

[28] Johnson R., Baker J. (1987). Assay of serum fructosamine: Internal vs. external standardization. Clin. Chem..

[29] Reznick A.Z., Packer L. (1994). Oxidative damage to proteins: Spectrophotometric method for carbonyl assay. Methods Enzymol..

[30] Ellman G.L. (1959). Tissue sulfhydryl groups. Arch. Biochem. Biophys..

[31] Borys J., Maciejczyk M., Krȩtowski A.J., Antonowicz B., Ratajczak-Wrona W., Jablonska E., Zaleski P., Waszkiel D., Ladny J.R., Zukowski P. (2017). The redox balance in erythrocytes, plasma, and periosteum of patients with titanium fixation of the jaw. Front. Physiol..

[32] Lushchak V.I. (2014). Free radicals, reactive oxygen species, oxidative stress and its classification. Chem. Biol. Interact..

[33] Poprac P., Jomova K., Simunkova M., Kollar V., Rhodes C.J., Valko M. (2017). Targeting free radicals in oxidative stress-related human diseases. Trends Pharmacol. Sci..

[34] Peluso I., Raguzzini A. (2016). Salivary and urinary total antioxidant capacity as biomarkers of oxidative stress in humans. Patholog. Res. Int..

[35] Cobley J.N., Fiorello M.L., Bailey D.M. (2018). 13 Reasons why the brain is susceptible to oxidative stress. Redox Biol..

[36] Maciejczyk M., Żebrowska E., Chabowski A. (2019). Insulin resistance and oxidative stress in the brain: What’s new?. Int. J. Mol. Sci..

[37] Aoyama K., Nakaki T. (2013). Impaired glutathione synthesis in neurodegeneration. Int. J. Mol. Sci..

[38] Johnson W.M., Wilson-Delfosse A.L., Mieyal J.J. (2012). Dysregulation of glutathione homeostasis in neurodegenerative diseases. Nutrients.

[39] Mandal P.K., Tripathi M., Sugunan S. (2012). Brain oxidative stress: Detection and mapping of anti-oxidant marker “Glutathione” in different brain regions of healthy male/female, MCI and Alzheimer patients using non-invasive magnetic resonance spectroscopy. Biochem. Biophys. Res. Commun..

[40] Sultana R., Piroddi M., Galli F., Butterfield D.A. (2008). Protein levels and activity of some antioxidant enzymes in hippocampus of subjects with amnestic mild cognitive impairment. Neurochem. Res..

[41] Dringen R., Hirrlinger J. (2003). Glutathione pathways in the brain. Biol. Chem..

[42] Liu Y., Hyde A.S., Simpson M.A., Barycki J.J. (2014). Emerging regulatory paradigms in glutathione metabolism. Adv. Cancer Res..

[43] Ott C., Jacobs K., Haucke E., Navarrete Santos A., Grune T., Simm A. (2014). Role of advanced glycation end products in cellular signaling. Redox Biol..

[44] Cai Z., Yan L.-J. (2013). Protein oxidative modifications: Beneficial roles in disease and health. J. Biochem. Pharmacol. Res..

[45] Tramutola A., Lanzillotta C., Perluigi M., Butterfield D.A. (2017). Oxidative stress, protein modification and Alzheimer disease. Brain Res. Bull..

[46] Maciejczyk M., Żebrowska E., Zalewska A., Chabowski A. (2018). Redox balance, antioxidant defense, and oxidative damage in the hypothalamus and cerebral cortex of rats with high fat diet-induced insulin resistance. Oxid. Med. Cell. Longev..

[47] Juranek J., Ray R., Banach M., Rai V. (2015). Receptor for advanced glycation end-products in neurodegenerative diseases. Rev. Neurosci..

[48] Morgan M.J., Liu Z.G. (2011). Crosstalk of reactive oxygen species and NF-κB signaling. Cell Res..

[49] Wautier M.-P., Chappey O., Corda S., Stern D.M., Schmidt A.M., Wautier J.-L. (2001). Activation of NADPH oxidase by AGE links oxidant stress to altered gene expression via RAGE. Am. J. Physiol. Metab..

